# No effects of siblings and twin testosterone transfer on autistic traits

**DOI:** 10.1002/jcv2.12069

**Published:** 2022-03-24

**Authors:** Melanie M. de Wit, Sander Begeer, Michel G. Nivard, Elsje van Bergen

**Affiliations:** ^1^ Department of Clinical, Neuro and Developmental Psychology Vrije Universiteit Amsterdam Amsterdam the Netherlands; ^2^ Amsterdam Public Health Research Institute Amsterdam the Netherlands; ^3^ Department of Biological Psychology Vrije Universiteit van Amsterdam and Amsterdam Public Health Research Institute Amsterdam the Netherlands; ^4^ LEARN! Research Institute Amsterdam the Netherlands

**Keywords:** autism spectrum disorders, siblings, testosterone, sex differences, child behaviour check list

## Abstract

**Background:**

Having twin and non‐twin siblings might influence autistic traits both prenatally and postnatally. The twin testosterone transfer hypothesis suggests that girls with a twin brother are exposed to higher levels of prenatal testosterone than girls with a twin sister, and that increased testosterone exposure masculinizes neural development and increases autistic traits. Postnatally, siblings may provide example behaviour, which could reduce autistic traits.

**Methods:**

We studied pre‐ and postnatal influences of twin and non‐twin siblings on mother and teacher‐reported autistic traits in 7714 dizygotic twins. We examined the effect of sex of the proband child and of the siblings. We fitted regression models (for boys and girls separately) with sex of co‐twin and having older and/or younger siblings of each sex as predictors.

**Results:**

Girls' mother‐reported autistic traits were slightly lower for those with a twin brother than those with a twin sister (*β* = −.08, *p* = .001, Cohen's *d* = −.13). This difference was not replicated in teacher‐reported autistic traits (*β* = .01, *p* = .734). Boys' (mother and teacher‐reported) autistic traits were not related to the sex of their co‐twin (*ps* > 0.50). Teacher‐reported autistic traits were slightly higher if girls had an older brother (*β* = .07, *p* = .013, Cohen's *d* = .12). Other than this small effect, we found no effect of non‐twin siblings on autistic traits in either girls or boys (*p*s > .18).

**Conclusions:**

We did not find increased autistic traits in girls with a twin brother compared to girls with a twin sister. This finding contributes to a body of literature that rejects the twin testosterone transfer hypothesis. In addition, we found little evidence for pre‐ and postnatal sibling influences. Our findings align with high heritability and absence of shared‐environmental influences in ASD.

## Graphical abstract

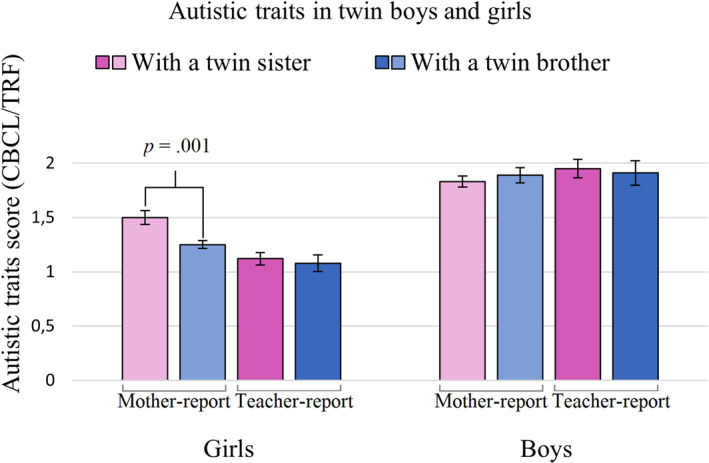

Leveraging a large twin sample (N∼7700), we did not find increased autistic traits in girls with a boy twin compared to girls with a girl twin. Our findings reject the hypothesis that testosterone is transferred prenatally between twins and/or that prenatal testosterone exposure increases children's autistic traits. In addition, children's autistic traits did not seem to be influenced by growing up with non‐twin siblings.

**Key Points**

- The twin testosterone transfer hypothesis proposes that exposure to testosterone increases autistic traits
- If testosterone transfers from male to female twins in utero, then girls with a twin brother (compared to a twin sister) should show increased autistic traits
- This study did not find increased autistic traits in girls with a twin brother
- The results suggest that testosterone may not be transferred prenatally, or does not influence autistic traits in later life
- The influence of siblings on children's autistic traits was also found to be negligible

## INTRODUCTION

Autism spectrum disorder (ASD) is a neurodevelopmental disorder characterized by atypical social‐communicative and repetitive and restrictive behaviour. The developmental course is influenced both by biological and environmental aspects (Tick et al., 2016). Twin siblings share their prenatal as well as their postnatal environment, and non‐twin siblings share their postnatal environment. Therefore, having siblings might be an important predictor of an individual's autistic traits. However, the influence of twin or non‐twin siblings remains poorly understood. Drawing on a large community sample of children, we studied pre‐ and postnatal influences of twin and non‐twin siblings on autistic traits and examine the effect of sex of the proband child and of the siblings.

It is still not fully understood why ASD is more common in boys than girls (Christensen et al., 2016). The extreme male brain theory hypothesizes that ASD is attributable to hypermasculinization of the brain (Baron‐Cohen, 2002; Werling & Geschwind, 2013). Hypermasculinization starts in the uterus and is driven by androgens, specifically testosterone (Auyeung et al., 2009). In previous research, several methods have been applied to test the association between testosterone exposure and ASD or autistic traits, but evidence of this association is mixed.

One method to study the association between testosterone and ASD or autistic traits is by directly measuring testosterone levels. Direct measures include prenatal testosterone levels in amniotic fluid and postnatal testosterone levels in blood or saliva. Some studies have found an association between testosterone levels in amniotic fluid and ASD or autistic traits (Auyeung et al., 2009, 2012; Baron‐Cohen et al., 2014), but others have not replicated these findings (Kung, Spencer, et al., 2016). Moreover, studies assessing testosterone concentrations in amniotic cord blood and early postnatal saliva did not find associations with ASD and autistic traits (Kung, Constantinescu, et al., 2016; Whitehouse et al., 2012). A recent meta‐analysis across three studies found a small but significant association between prenatally measured amniotic fluid testosterone with ASD diagnosis/autistic traits (*r* = .28, *p* < .001). However, this effect was only found for testosterone measured prenatally and was driven by only one lab that reported an association. This meta‐analysis also showed that testosterone measured peri‐ and postnatally was not associated with autistic traits (Coscini et al., 2021). However, the authors noted that measuring androgens is challenging for researchers and invasive for participating mothers and babies. Therefore, the studies included in their meta‐analysis were from few independent research groups and had a high potential for bias due to small and unrepresentative samples.

A second method to test influences of testosterone on autistic traits or ASD is by using indirect measures of testosterone exposure, for example, by assessing conditions that are linked to atypical sex hormone levels, physical characteristics related to testosterone, or differences between twins. One powerful approach to evaluate the effect of testosterone on autistic traits and ASD is to study females with congenital adrenal hyperplasia (CAH), a condition in girls that severely increases prenatal testosterone levels. Some studies have reported increased autistic traits or risk of ASD diagnosis in people with CAH (Engberg et al., 2015), whereas others have not (Kung, Spencer, et al., 2016). A recent meta‐analysis focussing on the comorbidity between conditions associated with atypical sex hormone levels, such as CAH, found no associations with ASD for most assessed conditions (odds ratios [OR] ranging from 1.10 to 1.55). However, some conditions linked to higher or lower levels of testosterone were associated with increased ASD risk, indicating a complex relationship between testosterone and ASD (May et al., 2021). Physical characteristics related to testosterone that have been studied for their relation with autistic traits are penile length and anogenital distance (the distance from the anus to the base of the scrotum in males and to the posterior fourchette in females; Boas et al., 2006; Thankamony et al., 2016). However, neither characteristic has been found to predict autistic traits at birth or 3 months of age (Kung et al., 2021).

Twin designs may be additionally promising in studying the effect of testosterone on ASD and autistic traits. The twin testosterone transfer (TTT) hypothesis suggests that (a) girls with a twin brother are exposed to higher levels of prenatal testosterone than girls with a twin sister (Even & Vom Saal, 1992; Miller, 1994), and (b) increased testosterone exposure leads to masculinized development and more autistic traits (Baron‐Cohen, 2002). The TTT hypothesis derives from research in rodents which shows that females that have been close to male littermates in the uterus develop more masculine features (Ryan & Vandenbergh, 2002). In a review study, the TTT hypothesis was considered biologically viable in humans, yet evidence of physiological (e.g., age at menarche, height, handgrip strength) and behavioural (e.g., alcohol use, eating disorders, sensation seeking) effects of testosterone transfer is inconsistent. The most consistent evidence from twin studies of an effect of testosterone on human behaviour applies to cognition, where cognitive functioning in girls with a boy twin shows a more masculine profile (e.g., increased performance on mental rotation tests, decreased communicative development; Ahrenfeldt et al., 2020). However, when directly measuring testosterone levels in umbilical cord blood at birth, Kuijper et al. (2015) found that having a boy (vs. a girl) twin did not increase testosterone levels.

Two studies that considered autistic traits in samples of over 6000 girls found results opposing the TTT hypothesis: girls with a twin sister showed more autistic traits than girls with a twin brother (Attermann et al., 2012; Eriksson et al., 2016). Eriksson et al. (2016) used a comprehensive measure of 17 autistic traits measured with the Autism—Tics, ADHD, and other Comorbidities inventory (A‐TAC). The results from Attermann et al. (2012) were however based on a limited, 5‐item set of the Child Behaviour Checklist (CBCL; Achenbach & Rescorla, 2001), items that mostly measured ADHD traits. Moreover, both these studies only used parent‐reported data, which might have resulted in biased results (see Constantino et al., 2007). Both Eriksson et al. (2016) and Attermann et al. (2012) propose that their results may stem from parental rater bias. Boys on average have more autistic traits than girls (Lai & Szatmari, 2020). In the presence of contrast effects, parents may under‐report autistic traits in daughters if they also have sons, because sons may influence their frame of reference. However, the absence of increased autistic traits in girls with a twin brother does add to the accumulating evidence against the TTT hypothesis.

In addition to prenatal influences, siblings might have an impact postnatally. Some research suggests that having siblings is beneficial in ASD treatment outcomes (de Veld et al., 2020; Ferraioli et al., 2012). Findings suggest, for example, that having older siblings helps children with ASD to develop social skills and theory of mind (Ben‐Itzchak et al., 2018; Matthews & Goldberg, 2016). While some research suggests that siblings influence social skills and treatment outcomes, little is known about their influences on the broad spectrum of autistic traits.

In conclusion, comprehensive research on sibling effects on autistic traits both biologically and environmentally is scarce and contradictory. The current study aimed to replicate and extend previous results regarding TTT and autistic traits by comparing a broad range of autistic traits in girls with a twin brother to girls with a twin sister. In addition, we addressed the potential issue of parental contrast effects by comparing parent and teacher reports. In line with the TTT hypothesis, we expected more autistic traits in girls with a twin brother than girls with a twin sister. To consider the impact of siblings on autistic traits, we compared boys with a twin sister to those with a twin brother, and explored the influence of non‐twin siblings. Furthermore, the study corrected for commonly used covariates associated with ASD risk to reduce possible confounding of results (i.e., parental educational attainment, birth weight, age, gestational age and school grades; Larsson et al., 2005). The study was preregistered on AsPredicted: 53015.

## MATERIALS AND METHODS

### Participants

Participants were 9‐ to 10‐year old twins from the Netherlands Twin Register (NTR; Ligthart et al., 2019). CBCL (Achenbach & Rescorla, 2001) data were available for a total of 14,973 individual dizygotic twin members. After exclusion of very prematurely born children, children with missing data on the autistic traits scale, outliers on the total autistic traits score (>4 SDs), or outliers on age (>3 SDs), 7714 individual participants remained. See Appendix S1 (Supporting Information S1) for participant details.

### Procedure

The NTR has been collecting longitudinal twin data on behavioural, health‐related, demographic, and biological factors in twins and multiples, and their families since 1985. Twins and their families were recruited either at birth or later in development and after providing informed consent mothers received the first questionnaire on pre‐ and perinatal variables including birth weight and gestational age. Parents continued to receive questionnaires every 2 to 3 years. At age 9–10 years, parents of twins received the CBCL (Achenbach & Rescorla, 2001), as well as additional questions on for example, demographics, and sibling constellation (Ligthart et al., 2019). With parent consent, the child's teacher was invited to fill out a survey including the teacher report form (TRF; Achenbach & Rescorla, 2001) and questions on school grades (van Bergen et al., 2018). Zygosity was determined using parent‐reported questionnaires (90%) or analysis of DNA (10%).

### Measures

Autistic traits were quantified as the sum of the scores of a validated subset (So et al., 2012) of 10 items from the (mother‐reported) CBCL and (teacher‐reported) TRF (Achenbach & Rescorla, 2001). The CBCL/TRF questionnaires measure challenging behaviour in children and adolescents during the past 6 months. They include 118 questions where respondents are asked to say to what extent the behaviour describes the child (0 = not true, 1 = somewhat or sometimes true, 2 = very true or often true). So et al. (2012) reported a Cronbach's alpha of 0.75 for a combined parent‐ and teacher‐report scale specifically, indicating a reliable measure. Although So et al. (2012) did not report sensitivity and specificity for the separate parent and teacher scales, they did report a sensitivity of 91% and a specificity of 84% for a combined parent‐ and teacher‐report scale. In our sample, Cronbach's alpha was 0.68 for the mother‐report and 0.74 for the teacher‐report scale. For analyses we applied a square root transformation for mother‐ and teacher‐reported autistic traits score because the data showed an L‐shaped distribution.

We measured several covariates. Teacher‐reported school performance for math, reading and language were scored [1 (fail), 2 (poor), 3 (satisfactory), 4 (above average) and 5 (good/excellent)]. Parental educational attainment was based on the educational level of both mother and father and ranged from ‘profession on unskilled level’ (1) to ‘profession on level scientific education’ (5). Birth weight (grams) and gestational age (weeks) were assessed in the first wave of NTR questionnaires.

### Statistical analyses

One random member of the dizygotic same‐sex twin pairs was taken into the analyses to account for dependency in the data. Descriptive statistics were calculated for the mother‐ and teacher‐reported autistic traits score for sex and sibling type. Separate one‐way ANOVAs were applied for all covariates to test for differences between boys and girls with a boy twin and boys and girls with a girl twin. If significant differences were found in any of these variables they were taken into further analyses as covariates.

Multiple regression analyses were used for boys and girls separately with co‐twin sex (boy or girl), younger brother, older brother, younger sister, older sister (all coded as 0 for no and 1 for yes), and included the significant covariates as predictors and the square root transformed mother‐reported and teacher‐reported autistic traits scores as dependent variables. In addition, stepwise regression analysis was performed to test whether a model where co‐twin sex was included added significantly to the explained variance of autistic traits compared to a model without co‐twin sex. Details are described in Appendix S1 (Supporting Information S1).

Following the pre‐registration, primary analyses focused on understanding the influence of having siblings on mother‐reported autistic traits in girls. All additional analyses were not preregistered and are therefore exploratory. Contrary to the preregistration, no descriptive statistics are provided for the siblings, since autistic traits were not assessed in siblings.

## RESULTS

A moderate correlation of *r* = .42 was observed between mother‐reported and teacher‐reported autistic traits. Descriptive statistics of mother‐ and teacher‐reported autistic traits per sex and sibling type are presented in Table 1. Overall, boys showed more autistic traits than girls on mother‐reported (*t* = 10.71, *df* = 7642.40, *p* < .001, Cohen's *d* = .24) and teacher‐reported (*t* = 11.44, *df* = 3366.63, *p* < .001, Cohen's *d* = .39). Further analyses were performed for boys and girls separately. Descriptive statistics of demographics are presented in Table S1 (Supporting Information S1).

**TABLE 1:** Descriptive statistics for parent‐ and teacher‐reported autistic traits per sex and sibling type

		***N***	***M***	***SD***	***N***	***M***	***SD***
		**Mother‐reported autistic traits**			**Teacher‐reported autistic traits**		
**Girls**							
	Twin brother	1,952	1.25	1.65	867	1.12	1.71
	Twin sister	958	1.50	1.91	450	1.08	1.62
	Older brother	759	1.29	1.83	338	1.26	1.83
	Older sister	697	1.25	1.65	341	0.98	1.50
	Younger brother	284	1.31	1.72	139	1.01	1.59
	Younger sister	241	1.41	1.83	118	1.33	2.08
**Boys**							
	Twin brother	1,029	1.89	2.24	477	1.91	2.42
	Twin sister	1,951	1.83	2.21	870	1.95	2.46
	Older brother	722	1.83	2.15	324	1.89	2.33
	Older sister	747	1.89	2.30	347	1.93	2.44
	Younger brother	268	2.00	2.42	133	1.95	2.49
	Younger sister	264	1.80	2.20	122	1.82	2.50

*Note: N*, number of participants; *M*, mean; *SD*, standard deviation.

### Girls

**FIGURE 1:**
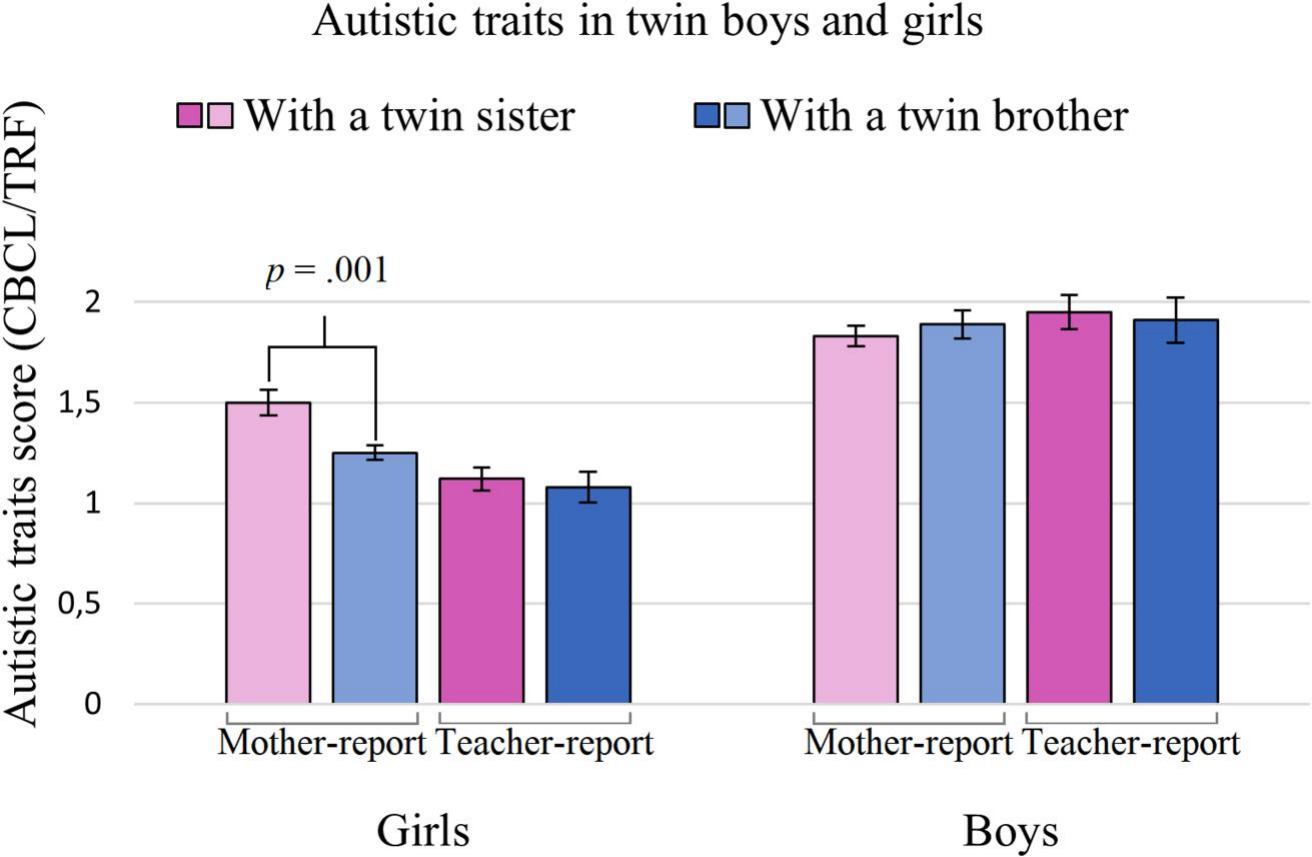
Mother‐ and teacher‐reported autistic traits in twin girls and boys with a twin brother and with a twin sister. Error bars represent standard error of the mean

One‐way ANOVAs showed no significant differences between girls with a twin sister and girls with a twin brother for all covariates (*p*s > .087), except for birth weight [*F* (1, 2865) = 6.64, *p* = .010, Cohen's *d* = .10]. Therefore, birth weight was included in the regression analyses. Multiple linear regression analysis showed fewer mother‐reported autistic traits in girls with a twin brother than girls with a twin sister (Cohen's *d* = −.13), but not teacher‐reported autistic traits (see Figure 1 and Table 2). For mother‐report, having non‐twin siblings of each sex did not significantly contribute to autistic traits. Birth weight showed a negative relation, where higher birth weight was associated with fewer mother‐reported autistic traits. In teacher‐report, having an older sister, younger brother, and younger sister did not significantly contribute to autistic traits, but having an older brother did (i.e., teachers reported more autistic traits if girls had an older brother compared to not having an older brother; Cohen's *d* = .12). Birth weight showed a negative relation, where higher birth weight was associated with fewer teacher‐reported autistic traits. Regression results are described in detail in Table 2. The results of stepwise regression are described in Appendix S2 (Supporting Information S1).

**TABLE 2:** Linear regression analysis for girls and boys for mother‐ and teacher‐reported autistic traits

	Mother‐report						Teacher‐report					
	*R* ^2^	*B*	*SE*	*β*	*t*	*p*	*R* ^2^	*B*	*SE*	*β*	*t*	*p*
**Girls**												
(Constant)	0.007*	1.74	0.12		14.07	<0.001	0.011	1.28	0.18		7.06	<0.001
Older Brother		−0.03	0.02	−0.02	1.16	0.247		0.09	0.04	0.07	2.48	0.013*
Older Sister		−0.02	0.03	−0.02	0.92	0.360		−0.05	0.04	−0.04	1.34	0.182
Younger Brother		−0.02	0.04	−0.01	0.62	0.534		−0.05	0.05	−0.03	1.07	0.284
Younger Sister		0.02	0.04	0.01	0.58	0.565		0.07	0.05	0.04	1.35	0.179
Birth weight		0.00	0.00	−0.05	2.40	0.016*		0.00	0.00	−0.06	2.02	0.043*
co‐twin sex (girl vs. boy)		−0.08	0.02	−0.06	3.33	0.001*		0.01	0.03	0.01	0.34	0.734
**Boys**												
(Constant)	0.001	1.41	0.04		37.92	<0.001	0.001	1.40	0.06	0.06	23.72	<0.001
Older Brother		−0.01	0.03	0.00	0.22	0.828		−0.01	0.045	0.05	0.13	0.898
Older Sister		0.01	0.03	0.01	0.40	0.691		−0.01	0.04	0.04	0.15	0.879
Younger Brother		0.04	0.04	0.02	0.93	0.351		0.02	0.06	0.06	0.23	0.821
Younger Sister		−0.03	0.04	−0.01	0.60	0.551		−0.06	0.07	0.07	0.86	0.388
co‐twin sex (girl vs. boy)		−0.01	0.01	−0.01	0.62	0.538		0.00	0.01	0.01	0.15	0.882

*Note*: Results marked with * are significant at *p* < 0.05.

### Boys

One‐way ANOVAs showed no significant differences between boys with a twin sister and boys with a twin brother for all covariates (*p*s > 0.164). Therefore, no covariates were included in the regression analysis for boys. Multiple linear regression analysis showed no effect of co‐twin sex on dizygotic boys for either mother‐ or teacher‐reported autistic traits. Having non‐twin siblings of each sex did not significantly contribute to mother and teacher‐reported autistic traits in boys. The regression results are described in Table 2. The results of stepwise regression are included in Appendix S2 (Supporting Information S1).

## DISCUSSION

This study examined pre‐ and post‐natal influences of twin and non‐twin siblings on autistic traits in a community sample of 9‐ and 10‐year‐olds. We tested the TTT hypothesis on autistic traits in girl twins and explored sibling influences on both girls and boys. The TTT hypothesis includes two propositions: (a) Testosterone is transferred prenatally and (b) testosterone has an effect on masculinization. This study utilised a twin‐design to test the TTT hypothesis, by assessing whether girls with a twin brother showed more autistic traits than girls with a twin sister. We found a small association of having a twin brother with decreased mother‐reported (and not teacher‐reported) autistic traits in girls. Decreased mother‐reported autistic traits in girls with a twin brother indicate a protective effect. Collectively, the findings contradict the TTT hypothesis. The protective effect in the mother data is in line with former studies (Attermann et al., 2012; Eriksson et al., 2016). In addition, the current study found that girls with an older brother showed increased teacher‐reported autistic traits, but the effect size was small. Other than this small effect, the study found no evidence of postnatal non‐twin sibling influences on autistic traits in girls or boys.

As our findings are inconsistent with the TTT hypothesis, we offer three alternative explanations. First, testosterone might not be transferred from male to female co‐twins in amounts that influence development. The assumption of prenatal testosterone exchange between twin siblings is predominantly based on animal studies, where it has been confirmed that females placed in between two male siblings in the womb become more masculine (Even & Vom Saal, 1992; Ryan & Vandenbergh, 2002). No concrete biological evidence has been provided to support prenatal testosterone transfer in humans (Kuijper et al., 2015). Moreover, studies exploiting (human) twin data to test the TTT have reported mixed results (Ahrenfeldt et al., 2020). While our findings indicate the absence of testosterone transfer between twins, there may be alternative explanations.

A second explanation could be that testosterone is transferred prenatally, but that testosterone exposure has no influence on autistic traits in later life. In line with this explanation, several recent studies have reported no association between either pre‐ or postnatal testosterone exposure and autistic traits or ASD (Kung et al., 2021; Kung, Constantinescu, et al., 2016; Kung, Spencer, et al., 2016; May et al., 2021). However, some studies did report associations between testosterone exposure and autistic traits or ASD (Auyeung et al., 2009, 2012; Baron‐Cohen et al., 2014). In all, evidence of the influence of testosterone exposure on autistic traits remains inconclusive. Recent reviews have suggested that additional research is needed to be able to draw stronger conclusions about the influence of testosterone on autistic traits (Coscini et al., 2021), and in line with Ahrenfeldt et al. (2020), we further suggest including non‐twin siblings more broadly in future research studies in twins to address the postnatal influence of having siblings.

Thirdly, current findings could in part be explained by rater contrast effects. While the first two reasons can explain the absence of a risk effect of having a boy co‐twin, they cannot explain the protective effect of having a boy co‐twin. Parents (of more than one child) are likely to rate a child's psychopathology relative to similarities and differences in their other child(ren). If one child exhibits many autistic traits, the symptoms of their other child(ren) may seem mild in comparison. Yet in absolute terms, the autistic symptoms could still be substantial (Eriksson et al., 2016). Because boys generally score higher on autistic traits than girls, parents might score girls with a twin brother lower because they compare their daughter to their son. This difference would explain the indicative findings of a protective effect of having a twin brother on mother‐reported autistic traits. However, we did not find increased autistic traits in boys with a twin sister, a finding that would also be expected under parental contrast effects. Compared with parents, teacher ratings may not be influenced as much by sibling effects, and teachers also have a broader reference frame of age‐appropriate behaviour. Some researchers have suggested, however, that teachers may be biased in different ways (e.g. twin confusion; Simonoff et al., 1998).

Furthermore, we found little evidence of postnatal non‐twin sibling influences on autistic traits. Teachers reported significantly increased autistic traits in girls with an older brother, but the effect size was small. Other than this effect, no sibling influences were found. Since we did not find any other non‐twin sibling influences on autistic traits, we speculate there may not be a true increase of autistic traits in girls with an older brother. The finding may have resulted from statistical error. In order to confirm this proposition, replication in future studies is desirable. In all, our results regarding non‐twin sibling influences contradict some previous studies that found that having an older sibling decreases autistic traits measured using parent‐, self‐ and teacher‐reports, assessment by healthcare professionals or test batteries in children with ASD and typically developing children (Ben‐Itzchak et al., 2018; Downey et al., 2013; McAlister & Peterson, 2013). These studies typically examined differences between only children and children with siblings. In our study in a twin sample, all children had by definition at least one sibling. To reconcile these findings, one possibility is that having more than one sibling does not additionally impact children's autistic traits.

Our findings align with the twin literature, where it is shown that sibling resemblance for ASD traits is due to shared genes, and not shared environments. Twin studies consistently show that individual differences in ASD traits mostly reflect genetic liability (heritability ∼80%; e.g., de Zeeuw et al., 2017; Polderman et al., 2015). Similarly, family studies show a strong familial risk of ASD, indicating high heritability (Xie et al., 2020). The remainder of individual differences in ASD traits is explained by environmental exposures that are unique to a child (i.e., non‐shared environmental influences). Twin studies do not find evidence for environmental effects shared by children growing up in the same family, which likely includes sibling effects. This study showed consistently that having siblings played little or no role in a child's autistic traits.

The major strength of the present study was the inclusion of both mother and teacher‐reported autistic traits in a large sample, which enabled us to address potential parental biases that were raised as a concern in previous studies (Attermann et al., 2012; Eriksson et al., 2016) There were, however, some limitations. First, we applied an indirect measure of testosterone exposure. Second, we assessed autistic traits using the CBCL which, despite its good psychometric properties for individuals with ASD, is not widely used to measure autistic traits (So et al., 2012).

## CONCLUSION

We examined the pre‐ and postnatal influences of having siblings on autistic traits in 9 to 10‐year‐old children. In line with previous studies on TTT and ASD, we did not find that having a twin brother, rather than a twin sister, increased autistic traits in girls. Our findings contribute to a body of research that rejects the TTT hypothesis (i.e., that testosterone is transferred prenatally, and increased prenatal testosterone increases autistic traits in later life). In addition, we found little evidence of non‐twin sibling influences postnatally. Our findings further suggest that parent reports on autistic traits in siblings might be biased, whereby parents exaggerate differences between siblings of the opposite sex. Our results align with the findings of high heritability of ASD and absence of shared‐environmental influences in twin and family studies. Our findings suggest that both pre‐ and postnatal sibling influences on autistic traits are negligible, and may be important in understanding the influence of siblings on ASD.

## CONFLICT OF INTERESTS

The authors have declared that they have no competing or potential conflicts of interest.

## ETHICAL CONSIDERATIONS

Informed consent was obtained from all participants. The study was approved by the Central Ethics Committee on Research Involving Human Subjects of the VU University Medical Centre, Amsterdam, an Institutional Review Board certified by the U.S. Office of Human Research Protections (IRB number IRB00002991 under Federal‐wide Assurance‐ FWA00017598; IRB/institute codes, NTR 03‐180).

## AUTHOR CONTRIBUTIONS

**Melanie de Wit**: Formal analysis, Investigation, Methodology, Project administration, Resources, Software, Writing—original draft. **Sander Begeer**: Conceptualization, Funding acquisition, Investigation, Methodology, Supervision, Writing—review & editing. **Michel Nivard**: Conceptualization, Funding acquisition, Investigation, Methodology, Resources, Supervision, Writing—review & editing. **Elsje van Bergen**: Conceptualization, Funding acquisition, Investigation, Methodology, Resources, Supervision, Writing—review & editing.

### OPEN RESEARCH BADGES

This article has earned a Preregistered Research Designs badge for having a preregistered research design, available at https://aspredicted.org/53qs8.pdf.

## Supporting information

Supporting Information S1

## Data Availability

Qualified researchers can request access to NTR data via the procedures outlined on the following website: https://tweelingenregister.vu.nl/submitting‐a‐data‐sharing‐request.
