# Supplementary material for: No effects of siblings and twin testosterone transfer on autistic traits
Source: JCPP Adv. 2022 Mar 24;2(1):e12069. doi: 10.1002/jcv2.12069 (PMC10242923; doi:10.1002/jcv2.12069)
Supplement: Supplementary file 1 — Supporting Information S1 [file JCV2-2-e12069-s001.docx]

**Supporting Information for:** No Effects of Siblings and Twin Testosterone Transfer on Autistic Traits by de Wit et al. (2022)

Contents

**[Appendix S1](#_Toc96671522)**[: Supplementary methods 1](#_Toc96671522)

[Participants 1](#_Toc96671523)

*[Exclusion criteria](#_Toc96671524)* [1](#_Toc96671524)

*[Zygosity](#_Toc96671525)* [1](#_Toc96671525)

[Measures: 2](#_Toc96671526)

[Statistical analyses: 2](#_Toc96671527)

**[Appendix S2:](#_Toc96671528)** [Supplementary results 2](#_Toc96671528)

[Girls: 2](#_Toc96671529)

[Boys: 2](#_Toc96671530)

[Tables 3](#_Toc96671531)

[Table S1 3](#_Toc96671532)

[References 5](#_Toc96671533)

# **Appendix S1**: Supplementary methods

## Participants

### *Exclusion criteria*

CBCL data were available for a total of 14,973 individual dizygotic twin members (3,791 boys with a boy twin, 3,800 boys with a girl twin, 3,581 girls with a girls twin, and 3,800 girls with a boy twin). Very prematurely born participants (< 33 weeks) were excluded from analysis since prematurity may increase ASD risk (Allen et al., 2020) and twins generally have high rates of prematurity (Büscher et al., 2000). After exclusion of very prematurely born children (41.2%), children that had missing data on at least one of the items of the autistic traits scale (10.6%), outliers on the total autistic traits score (> 4 SD’s) (0.2%) or outliers on age (> 3 SD’s) (0%), 7,714 individual (1969 boys with a boy twin, 1,957 boys with a girl twin, 1834 girls with a girl twin, and 1,954 girls with a boy twin) participants remained.

### *Zygosity*

In 90% of the same-sex twins, zygosity was determined using a parent-report questionnaire consisting of ten items on twin similarity. Zygosity based on this questionnaire has an accuracy of 93% when compared to zygosity based on DNA (Rietveld et al., 2000). In 10% zygosity was obtained from a comparative analysis of DNA.

## Measures:

Square root transformation was applied to the data since the mother- and teacher-reported autistic traits score had an L-shaped distribution (mother-report: skewness 2.24, kurtosis 7.27, teacher-report: skewness 2.17, kurtosis 5.94 ). This transformation resulted in skewness of 1.02 and kurtosis of .84 for mother-reported autistic traits and skewness of 1.11 and kurtosis of .77 for teacher-reported autistic traits.

## Statistical analyses:

Additional to multiple regression analyses described in the main text, stepwise linear regression was performed to determine the effect of co-twin sex beyond that of sibling constellation and covariates.

# **Appendix S2:** Supplementary results

## Girls:

Stepwise regression showed that co-twin sex predicted mother-reported autistic traits above and beyond birth weight and sibling constellation, *F* change = 11.08, *R^2^* change = .004, *p* = .001, but not teacher-reported autistic traits, *F* change = 0.12, *R^2^* change = .00, *p* = .734.

## Boys:

Stepwise regression analysis showed that co-twin sex does not significantly add to the model for neither mother-reported autistic traits, *F* change = 0.38, *R^2^* change = .00, *p* = .538) nor teacher-reported autistic traits, *F* change = 0.02, *R^2^* change = .00, *p* = .882).

# Tables

Table S1**.** Descriptive Statistics of Parental Educational Attainment, Age, Birth Weight, Gestational Age and School Grades per Sex and Twin Type.

|  |  | *N* | *M* | *SD* |
| --- | --- | --- | --- | --- |
| **Girls** |  |  |  |  |
| Parental educational attainment | Twin brother | 1,932 | 3.47 | .99 |
|  | Twin sister | 951 | 3.47 | 1.00 |
|  | All | 2,883 | 3.47 | .99 |
| Age | Twin brother | 1,952 | 9.85 | .46 |
|  | Twin sister | 958 | 9.85 | .46 |
|  | All | 2,910 | 9.85 | .46 |
| Birth weight (grams) | Twin brother | 1,922 | 2,537 | 463 |
|  | Twin sister | 945 | 2,585 | 466 |
|  | All | 2,867 | 2,553 | 465 |
| Gestational age (weeks) | Twin brother | 1,952 | 37.06 | 1.79 |
|  | Twin sister | 958 | 37.14 | 1.76 |
|  | All | 2,910 | 37.09 | 1.78 |
| School grade reading | Twin brother | 757 | 3.89 | 1.15 |
|  | Twin sister | 387 | 3.97 | 1.18 |
|  | All | 1,144 | 3.91 | 1.16 |
| School grade math | Twin brother | 856 | 3.65 | 1.22 |
|  | Twin sister | 444 | 3.77 | 1.20 |
|  | All | 1,300 | 3.69 | 1.22 |
| School grade language | Twin brother | 811 | 3.92 | .99 |
|  | Twin sister | 427 | 3.97 | .99 |
|  | All | 1,238 | 3.94 | .99 |
| **Boys** |  |  |  |  |
| Parental educational  attainment | Twin brother | 1,026 | 3.45 | 1.00 |
|  | Twin sister | 1,933 | 3.47 | .98 |
|  | All | 2,959 | 3.47 | .99 |
| Age | Twin brother | 1,029 | 9.86 | .46 |
|  | Twin sister | 1,951 | 9.85 | .46 |
|  | All | 2,980 | 9.85 | .46 |
| Birth weight  (grams) | Twin brother | 1,016 | 2,657 | 486 |
|  | Twin sister | 1,920 | 2,672 | 491 |
|  | All | 2,936 | 2,666 | 489 |
| Gestational age  (weeks) | Twin brother | 1,029 | 37.06 | 1.76 |
|  | Twin sister | 1,951 | 37.06 | 1.81 |
|  | All | 2,980 | 37.06 | 1.79 |
| School grade  reading | Twin brother | 417 | 3.55 | 1.27 |
|  | Twin sister | 761 | 3.65 | 1.25 |
|  | All | 1,178 | 3.62 | 1.25 |
| School grade math | Twin brother | 468 | 4.02 | 1.08 |
|  | Twin sister | 868 | 4.04 | 1.10 |
|  | All | 1,336 | 4.03 | 1.09 |
| School grade  language | Twin brother | 4,44 | 3.66 | 1.10 |
|  | Twin sister | 809 | 3.75 | 1.09 |
|  | All | 1,253 | 3.72 | 1.09 |

*Note. N* = Number of participants, *M* = Mean, *SD* = Standard Deviation.

# References

Allen, L., Leon-Attia, O., Shaham, M., Shefer, S., & Gabis, L. V. (2020). Autism risk linked to prematurity is more accentuated in girls. *PLOS ONE*, *15*(8), e0236994. https://doi.org/10.1371/JOURNAL.PONE.0236994

Büscher, U., Horstkamp, B., Wessel, J., Chen, F. C. K., & Dudenhausen, J. W. (2000). Frequency and significance of preterm delivery in twin pregnancies. *International Journal of Gynecology & Obstetrics*, *69*(1), 1–7. https://doi.org/10.1016/S0020-7292(99)00223-4

Rietveld, M., van der Valk, J., Bongers, I., Stroet, T., Slagboom, P., & Boomsma, D. (2000). Zygosity diagnosis in young twins by parental report. *Twin Research (2000)*, *3*(3), 134–141. https://doi.org/10.1375/136905200320565409
